# Severe Mitral Regurgitation in Mirror-Image Dextrocardia

**DOI:** 10.1016/j.jaccas.2025.104651

**Published:** 2025-08-06

**Authors:** Jinxia Zhang, Panguo Zhao, Feng Long, Xiaoliang Fu, Ying Zhang, Yangxin Chen, Dingcheng Xiang

**Affiliations:** a Department of Cardiology, General Hospital of Southern Theatre Command, PLA, Guangzhou, China; b Ultrasound Diagnosis Department, General Hospital of Southern Theatre Command, PLA, Guangzhou, China; c Department of Cardiology, Sun Yat-sen Memorial Hospital, Sun Yat-sen University, Guangzhou, China

**Keywords:** case report, mirror-image dextrocardia, MitraClip, mitral regurgitation, TEER, transcatheter mitral valve repair

## Abstract

**Background:**

Mirror-image dextrocardia with severe mitral regurgitation (MR) is a rare congenital anomaly that significantly increases the complexity of transcatheter mitral valve edge-to-edge repair (TEER).

**Case Summary:**

We report the case of a 72-year-old woman with mirror-image dextrocardia and severe MR who underwent successful TEER using the MitraClip system. Because of the anatomic reversal, several procedural adaptations were required, including modified transesophageal echocardiography views, adjustments to atrial septal puncture technique, and novel strategies for delivering the clip and resolving the “aortic hug.”

**Discussion:**

This case highlights the unique technical challenges of performing TEER in a patient with mirror-image dextrocardia. Specific procedural modifications were necessary for optimal catheter navigation and leaflet grasping.

**Take-Home Message:**

TEER in patients with mirror-image dextrocardia and severe MR requires specific adaptations. Successful outcomes require operators with substantial TEER experience and excellent 3-dimensional spatial reasoning.

## Graphical Abstract

**Figure:**
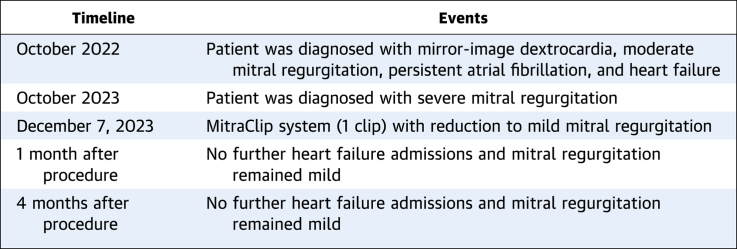

Transcatheter mitral valve edge-to-edge repair (TEER) has become an important treatment option for patients with mitral regurgitation (MR) who are at high surgical risk.^1^, ^2^, ^3^ Mirror-image dextrocardia, with an estimated prevalence of 1 in 10,000, is an extremely rare congenital cardiac positional anomaly that significantly increases the procedural complexity of TEER. Here, we report a case of mirror-image dextrocardia with severe functional MR successfully treated using the MitraClip (Abbott) system. We describe the procedural adaptations and strategies used, aiming to provide insights for future interventions in patients with similar anatomic challenges.

**Take-Home Messages**

• TEER in patients with mirror-image dextrocardia and severe MR requires specific adaptations, including modified TEE views, atrial septal puncture techniques, and strategies to resolve anatomic obstacles such as the “aortic hug.”
• Successful outcomes require operators with substantial TEER experience and excellent three-dimensional spatial reasoning.

## History of Presentation

A 72-year-old woman presented with recurrent dyspnea and shortness of breath since 2019. Coronary angiography showed no significant coronary artery stenosis. She was treated with guideline-directed medical therapy, but her symptoms persisted and progressively worsened. In October 2023, transthoracic echocardiography revealed severe MR.

## Past Medical History

**Figure 1:**
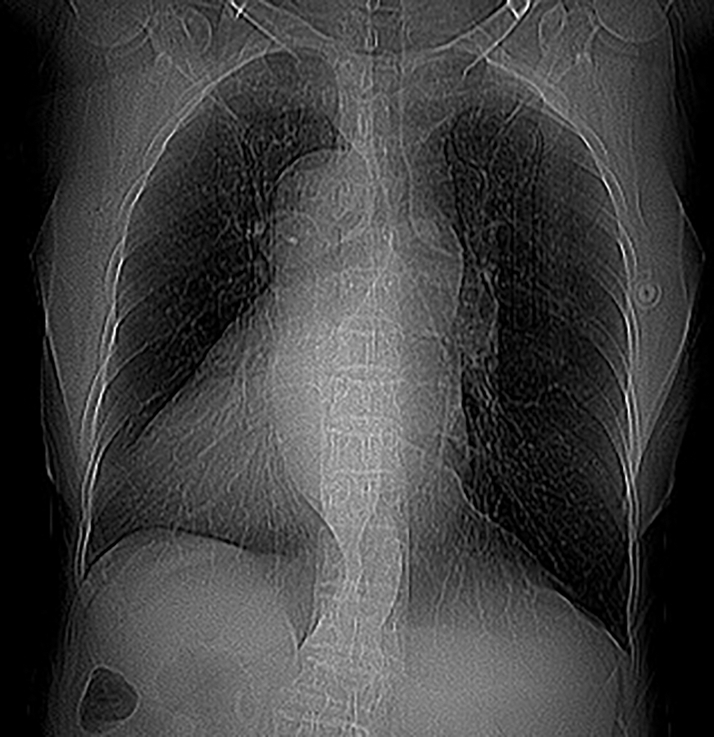
Chest X-Ray Demonstrating Mirror-Image Dextrocardia

In October 2022, the patient was diagnosed with mirror-image dextrocardia (Figure 1), moderate MR, persistent atrial fibrillation, and heart failure with a left ventricular ejection fraction of 44%.

## Investigations

In November 2023, the patient was referred to our tertiary care center. Electrocardiography confirmed atrial fibrillation. Transesophageal echocardiography (TEE) revealed severe MR originating primarily between A2 and P2 segments. The mitral annulus measured 45 mm in diameter. The lengths of the anterior and posterior mitral leaflets were 24 mm and 10 mm, respectively. The vena contract width was approximately 5.5 mm, and the effective regurgitant orifice area was estimated at 11 cm^2^. Her MitraScore^4^ was 0, and her Society of Thoracic Surgeons predicted mortality risk was 11.4%.

## Management

The patient declined surgical mitral valve repair. After multidisciplinary discussion, TEER was recommended as an alternative. Following a comprehensive preoperative assessment and simulation-based training, the TEER procedure was performed on December 7, 2023.

**Figure 2:**
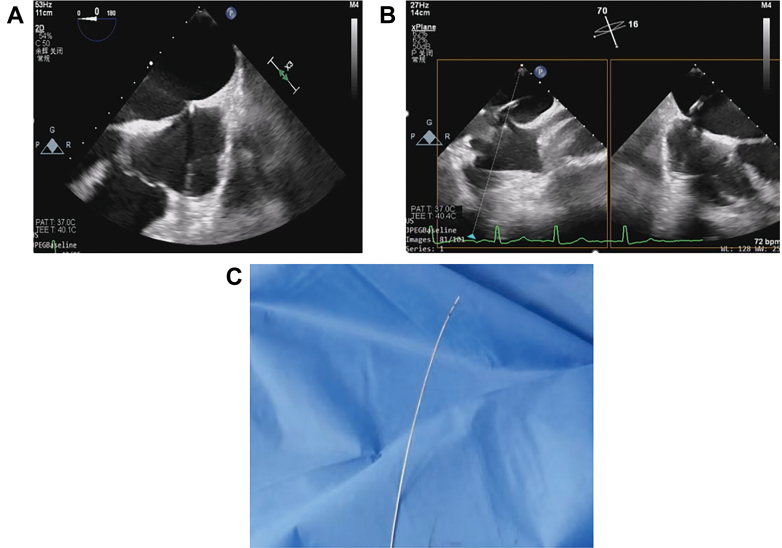
Transesophageal Echocardiography Images of Atrial Septal Puncture and Reverse-Shaped Puncture Needle (A) Measurement of atrial septal height at 0° in the reversed 4-chamber view. (B) Atrial septal puncture at 70° in the 2-chamber view. (C) Modified straightened configuration of the atrial septal puncture needle.

Access was obtained via the left femoral vein. TEE was employed to guide atrial septal puncture, with septal height measured at 0° in the reversed 4-chamber view and puncture performed at 70° in the 2-chamber view (Figures 2A and 2B). Multiple puncture attempts failed to produce a satisfactory tenting sign, despite using both standard and increased curvature approaches. Eventually, by straightening the transseptal needle (Figure 2C), we achieved a satisfactory tenting sign and performed the puncture at a site 4 cm superior to the mitral annulus (Video 1, Video 2, Video 3).

**Figure 3:**
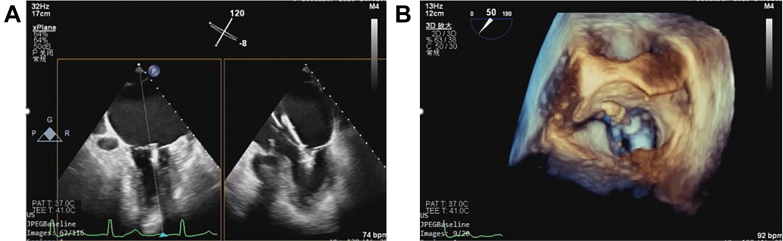
TEE Images Illustrating the Aortic Hug Phenomenon (A) Two-dimensional transesophageal echocardiography (TEE) images. (B) Three-dimensional TEE image.

After advancing the steerable guide catheter (SGC), we noted atypical responses during rotation—clockwise rotation advanced the SGC, contrary to usual anatomic configurations. Upon appropriate orientation, the clip delivery system (CDS) was introduced. Given the patient's functional MR and a 10-mm posterior leaflet, we initially selected an NTR clip. However, the “blue-to-blue” advancement led to an “aortic hug,” with the clip positioned too close to the aortic valve (Figure 3). Multiple attempts to resolve this using P and M key adjustments and SGC rotation were unsuccessful. Despite entering the left ventricle and attempting leaflet capture, control over the anterior mitral leaflet was inadequate (Video 4).

We then switched to the XTR clip for increased reach. Following a previously reported strategy,^5^ we attempted to advance the CDS into the SGC by rotating counterclockwise 90°, but the aortic hug persisted. We then employed a novel approach: rotating the CDS clockwise 90° before insertion. We intentionally induced the aortic hug via the P key, then moved the SGC away from the aorta using counterclockwise rotation. By increasing the + key to advance the clip anteriorly and medially, and decreasing the P key to reposition the clip toward zone 1, we achieved optimal alignment. TEE confirmed appropriate axial orientation.

**Figure 4:**
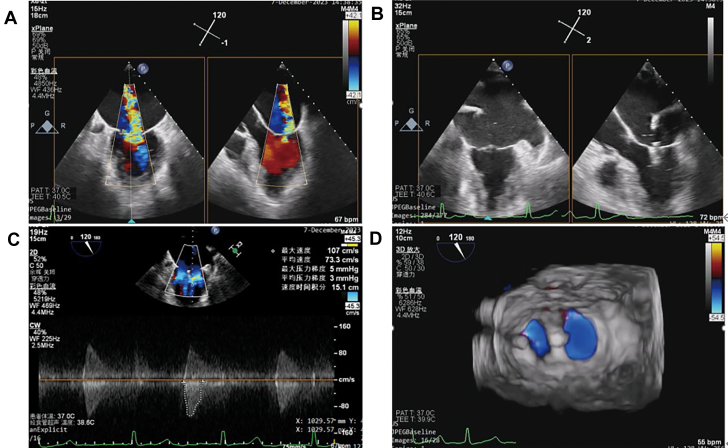
Transesophageal Echocardiography Images Obtained During the Transcatheter Mitral Valve Edge-to-Edge Repair Procedure (A) Evaluation of severe mitral regurgitation intraoperatively. (B) Successful grasping of the anterior and posterior mitral valve leaflets. (C) Measurement of mitral valve flow velocity and mean pressure gradient. (D) Visualization of the tissue bridge structure and residual regurgitation.

A grasp test was performed, clip arms were opened, and under three-dimensional TEE guidance, the clip arm orientation was aligned with the mitral commissures. After insertion into the left ventricle, several adjustments were made at 120° (bicommissural view) to successfully grasp both anterior and posterior mitral leaflets. The CDS and delivery system were then withdrawn. The procedure lasted approximately 4 hours and was completed successfully (Figure 4, Videos 5 and 6).

## Outcome and Follow-Up

Post-procedure two-dimensional TEE demonstrated a significant reduction in MR. The anterior and posterior mitral leaflet lengths at the grasping site were 10 mm and 8 mm, respectively. The mean transmitral pressure gradient was 3 mm Hg. Three-dimensional TEE confirmed the presence of a stable tissue bridge between the leaflets.

**Figure 5:**
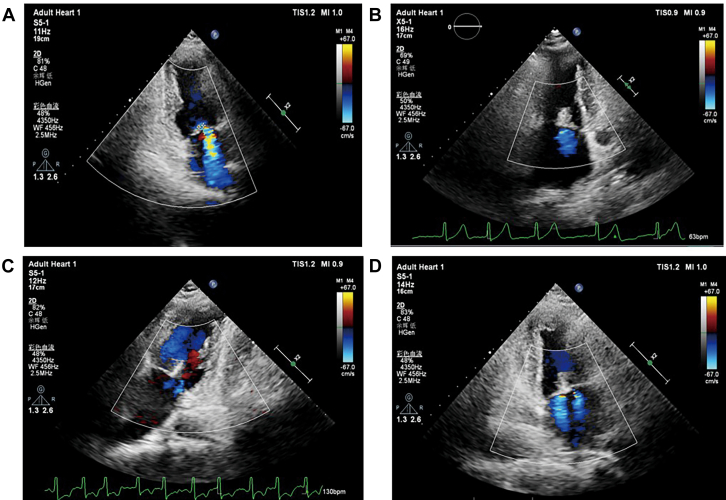
Transthoracic Echocardiography Images Obtained During Follow-Up (A) Before transcatheter mitral valve edge-to-edge repair (TEER). (B) Postoperative day 2. (C) One month after TEER. (D) Four months after TEER.

Transthoracic echocardiography on postoperative day 2 showed only mild residual MR. The total area of the mitral valve double orifice was 2.8 cm^2^. The patient reported substantial symptomatic relief. One week later, her 6-minute walk test distance reached 388 m without discomfort. Follow-up assessments at 1 and 4 months confirmed stable clinical and echocardiographic outcomes (Figure 5).

## Discussion

Severe MR in the setting of mirror-image dextrocardia is exceptionally rare. To date, only 2 prior cases of TEER in patients with mirror-image dextrocardia have been reported. One case resulted in death owing to delayed clip detachment complications,^6^ whereas a moderate residual MR outcome was achieved in the other case.^5^ Real-world experience and procedural knowledge in this context remain limited.

According to current guidelines, both surgical repair and TEER are viable options for functional MR.^1^^,^^2^ In this case, TEER was chosen based on several factors, as follows: 1) the patient's firm refusal of surgery, 2) the operator's extensive experience with TEER (>100 successful cases), and 3) the patient's elevated surgical risk.

This procedure presented several unique technical challenges and key takeaways:

1. TEE-guided septal puncture: Ideal TEE imaging was difficult to achieve. We measured the septal height in a reversed 4-chamber view at 0° and performed the puncture in a 2-chamber view at 70°.
2. Septal puncture technique: Initial puncture attempts with both standard and increased needle curvature failed to show a satisfactory tenting sign. Straightening the needle ultimately produced a satisfactory tenting sign and enabled successful puncture.
3. Catheter manipulation: In this patient, clockwise rotation of the SGC advanced the tip, contrary to typical cases. Adjustments required mirror-image logic.
4. Clip delivery strategy: Owing to the aortic hug phenomenon encountered when using the standard blue-to-blue approach, we rotated the CDS 90° clockwise for successful insertion.
5. Control key functions: Key movements differed from standard anatomy. The +key advanced the clip medially (valve interior), the −key moved it laterally. The P and M keys advanced and retracted the clip, respectively.
6. Gentle technique: Owing to anatomic variation, cautious and deliberate manipulation was critical to avoid complications such as chordal entanglement, leaflet injury, cardiac perforation, and pericardial tamponade.

**Visual Summary:** Timeline of the Case

Timeline	Events
October 2022	Patient was diagnosed with mirror-image dextrocardia, moderate mitral regurgitation, persistent atrial fibrillation, and heart failure
October 2023	Patient was diagnosed with severe mitral regurgitation
December 7, 2023	MitraClip system (1 clip) with reduction to mild mitral regurgitation
1 month after procedure	No further heart failure admissions and mitral regurgitation remained mild
4 months after procedure	No further heart failure admissions and mitral regurgitation remained mild

## Conclusions

We provided a detailed account of the customized TEER approach in a patient with mirror-image dextrocardia. The procedural modifications and lessons learned may serve as a practical reference for clinicians encountering similar anatomic challenges.

## Funding Support and Author Disclosures

The authors have reported that they have no relationships relevant to the contents of this paper to disclose. This work was supported by research grants from the 10.13039/501100010256Guangzhou Science and Technology Plan Project (2023B01J1011).

## Appendix

Video 1 Preoperative TEE Indicates Significant Mitral Regurgitation

Video 2 The 3-D TEE Before Operation

Video 3 Successfully Punctured the Atrial Septum

Video 4 The “Aorta Hug”

Video 5 Successfully Captured Two Leaflets of Mitral Valve After Adjustment

Video 6 The 3-D TEE After Captured
